# Supplementary material for: Integrative analysis reveals a conserved role for the amyloid precursor protein in proteostasis during aging
Source: Nat Commun. 2023 Nov 3;14:7034. doi: 10.1038/s41467-023-42822-1 (PMC10624868; doi:10.1038/s41467-023-42822-1)
Supplement: Supplementary file 1 — Supplementary Information [file 41467_2023_42822_MOESM1_ESM.pdf]

Supplementary Information

Integrative analysis reveals a conserved role for the amyloid precursor protein in proteostasis during aging

Vanitha Nithianandam,[1,2] Hassan Bukhari,[1,2] Matthew J. Leventhal,[3,4] Rachel A. Battaglia,[1,2] Xianjun Dong,[2,5] Ernest Fraenkel,[3] Mel B. Feany[1,2*]

[1]Department of Pathology, Brigham and Women's Hospital, Harvard Medical School, Boston, Massachusetts, USA.

[2]Aligning Science Across Parkinson's (ASAP) Collaborative Research Network, Chevy Chase, MD, 20815

[3]Department of Biological Engineering, Massachusetts Institute of Technology, Cambridge, MA, USA.

[4]MIT Ph.D. Program in Computational and Systems Biology, Cambridge, MA, USA.

[5]Genomics and Bioinformatics Hub, Brigham and Women's Hospital, Boston, MA, USA.

*****Correspondence to:

Mel B. Feany

*e-mail: mel_feany@hms.harvard.edu

This PDF file includes:

Supplementary Figures 1-10

Supplementary Figure 1

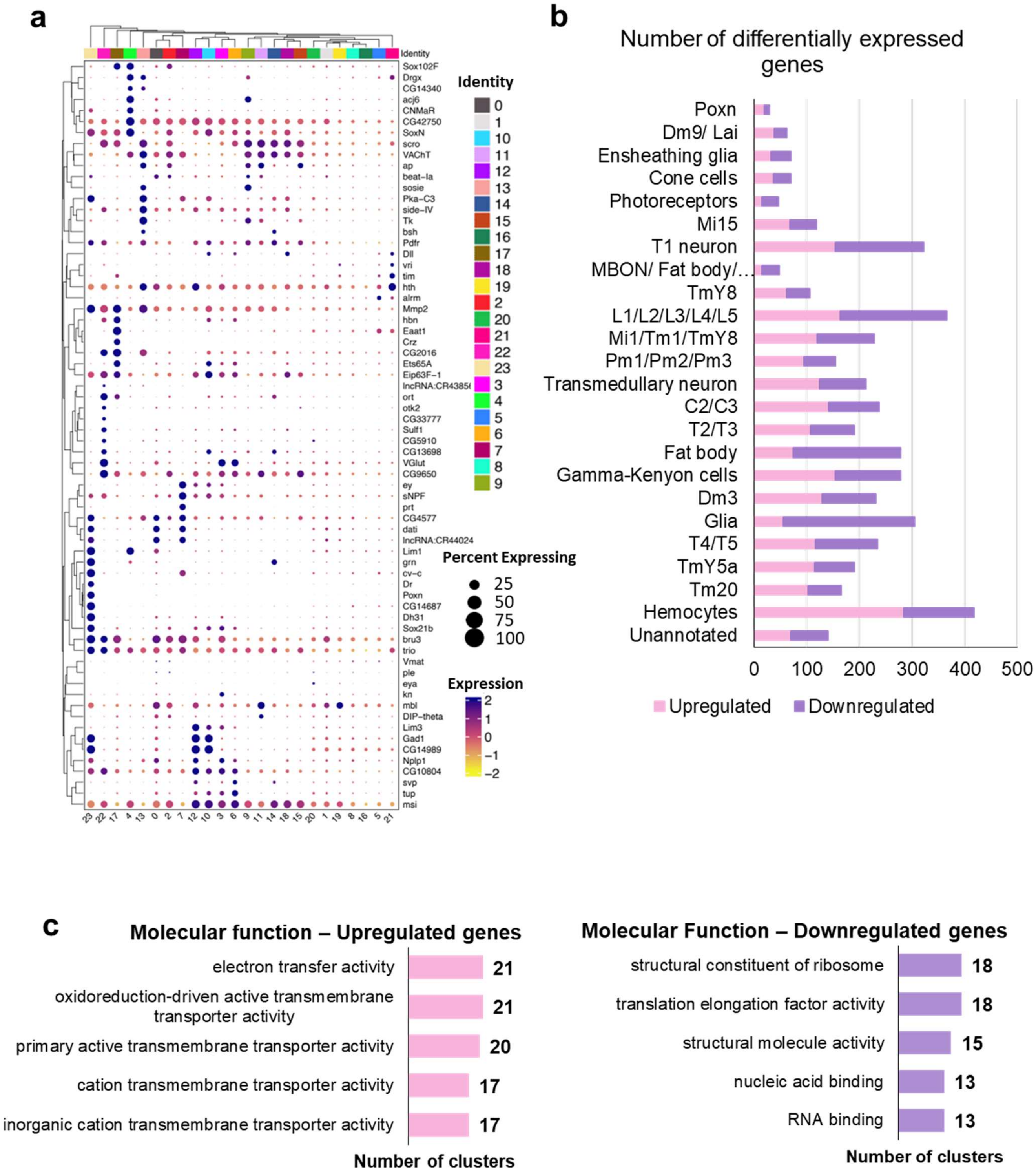

Supplementary Figure 1: a The dot plot shows the differential expression of marker genes in specific cell clusters. The X-axis and identity numbers indicate the cluster numbers as given in Figure **2b**. Percent expressing shows the percentage of cells expressing the marker in a cluster. The color of the dots indicates the expression level. **b** The bar graph shows the number of genes differentially expressed in the different cell populations due to the loss of Appl. Pink indicates the number of upregulated genes, and violet indicates the number of downregulated genes. **c** Gene ontology analysis of upregulated and downregulated genes from scRNA-seq analysis. Source data are provided as a Source Data file.

Supplementary Figure 2

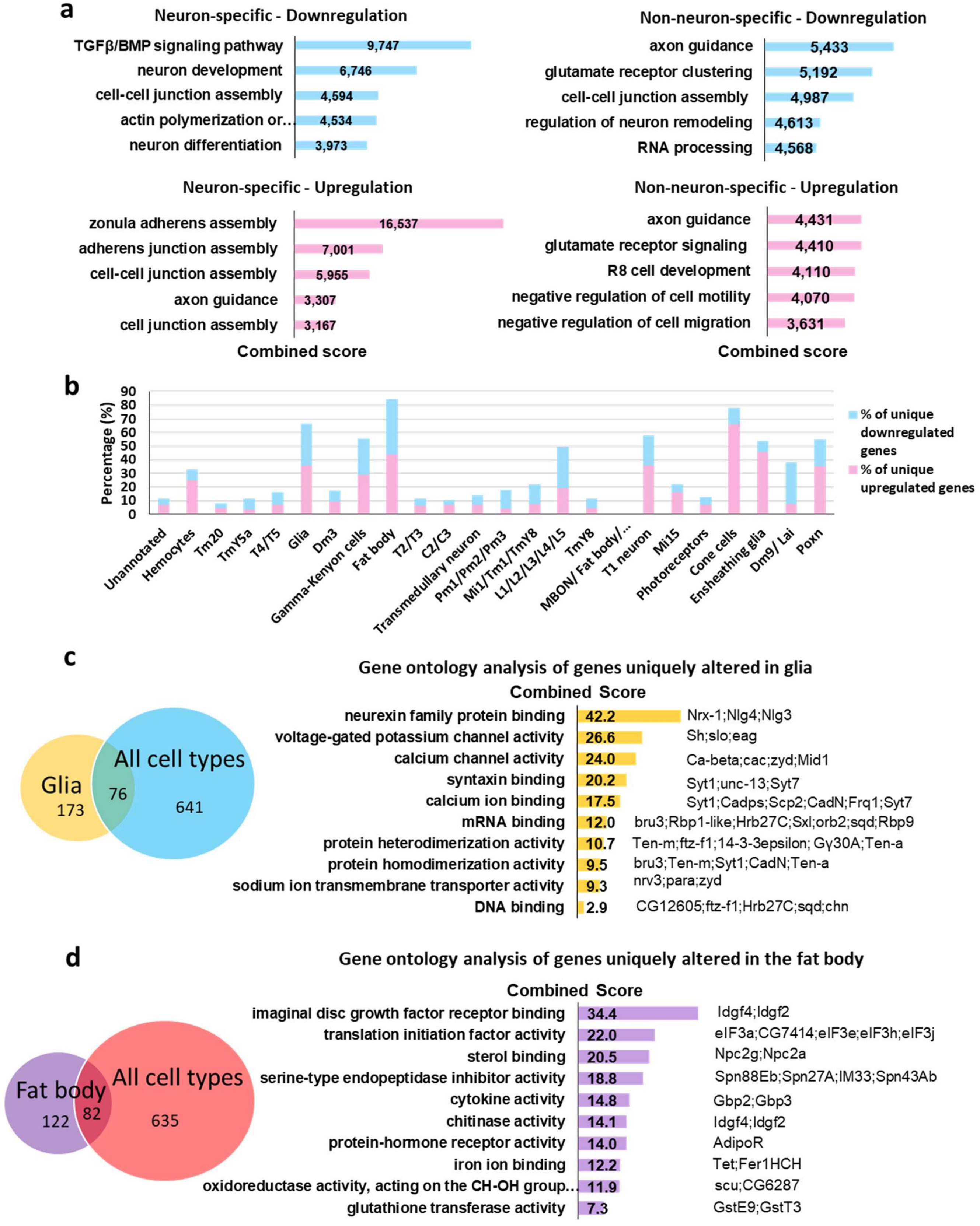

Supplementary Figure 2: Gene expression changes in the neuronal and non-neuronal populations of *Appl*¯ fly brains. a Gene ontology analysis of genes specifically altered in neuronal and non-neuronal populations categorized under biological process using FlyEnrichr. **b** The bar graph shows the percentage of genes uniquely altered in the different cell populations. **c** and **d** Gene ontology analysis of genes uniquely altered in glia and fat body categorized under molecular function using FlyEnrichr. The Venn diagram shows the number of regulated genes distinct and common in glia and fat body compared to other cell populations. In **a**, **c**, and **d,** the indicate the combined score (c = ln(p) * z), calculated using the p-value and z-score. The p-value is computed using Fisher's exact test, a binomial proportion test assuming a binomial distribution. Source data are provided as a Source Data file.

Supplementary Figure 3

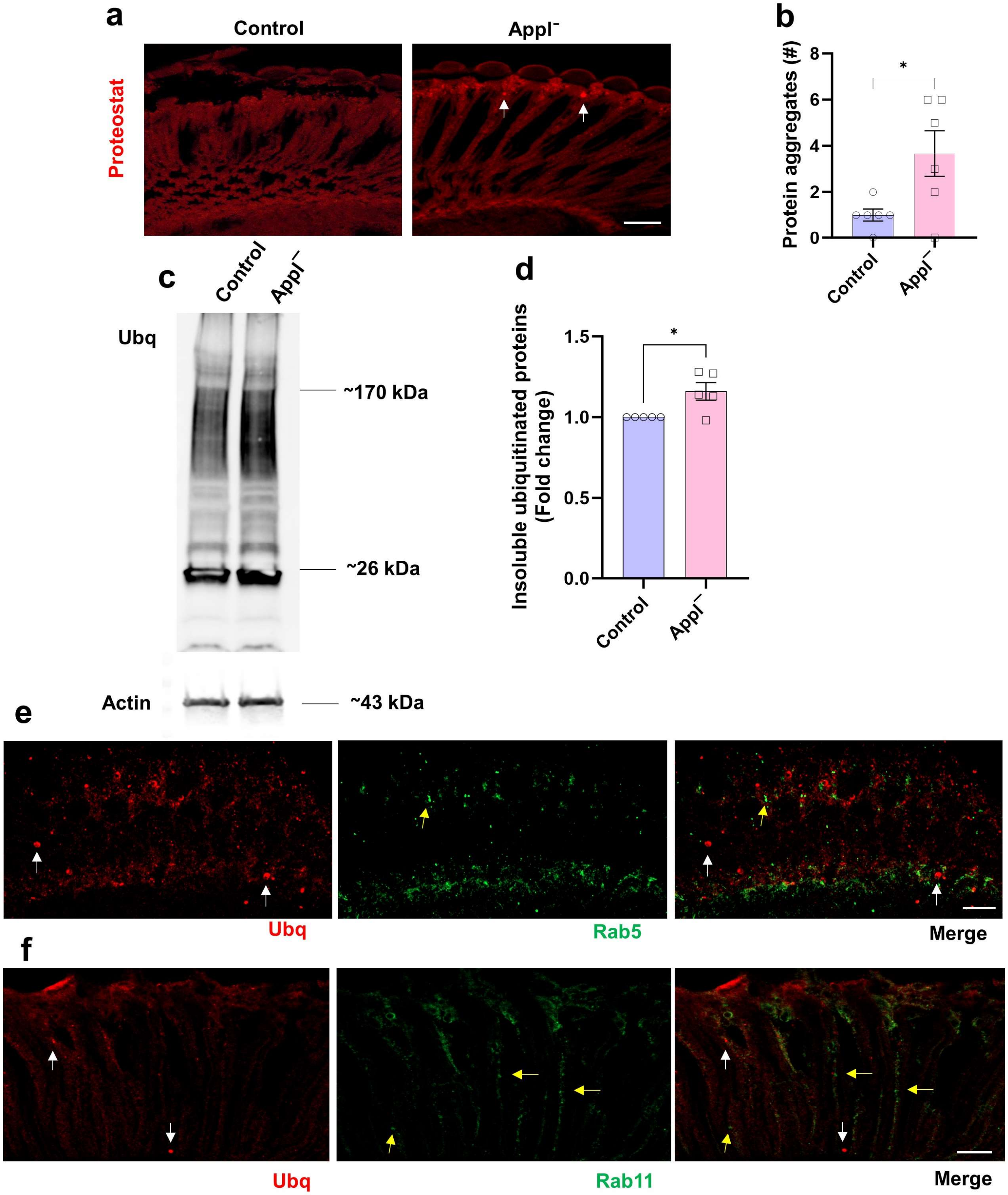

Supplementary Figure 3: Appl loss leads to protein aggregate formation. a Representative images showing protein aggregates (arrows) stained with the ProteoStat dye. **b** Quantification shows an increase in the number of protein aggregates in retinal sections of *Appl*$^{-}$ flies compared to controls. n = 6 per genotype. p value = 0.0401. **c,d** Western blot **(c)** and quantification **(d)** demonstrate an increase in the insoluble protein aggregates in *Appl*$^{-}$ flies compared to the controls. n = 5 repeats per genotype. Six males and six females per repeat. p value = 0.0434. **e,f** Ubiquitin-positive aggregates (white arrow, red) do not colocalize with the early endosomal marker, Rab5 (yellow arrow, green) or the recycling endosomal marker, Rab11 (yellow arrow, green) in *Appl*$^{-}$ flies. **(e, f)**. Control, *nSyb-GAL4/+* (**a,b**) and w^{1118} (**c,d**). *p<0.05, two-tailed Student's t-test **(b, d)**. Data are represented as mean ± SEM. Scale bars,10 µm. Flies are 10 days old. Source data are provided as a Source Data file.

Supplementary Figure 4

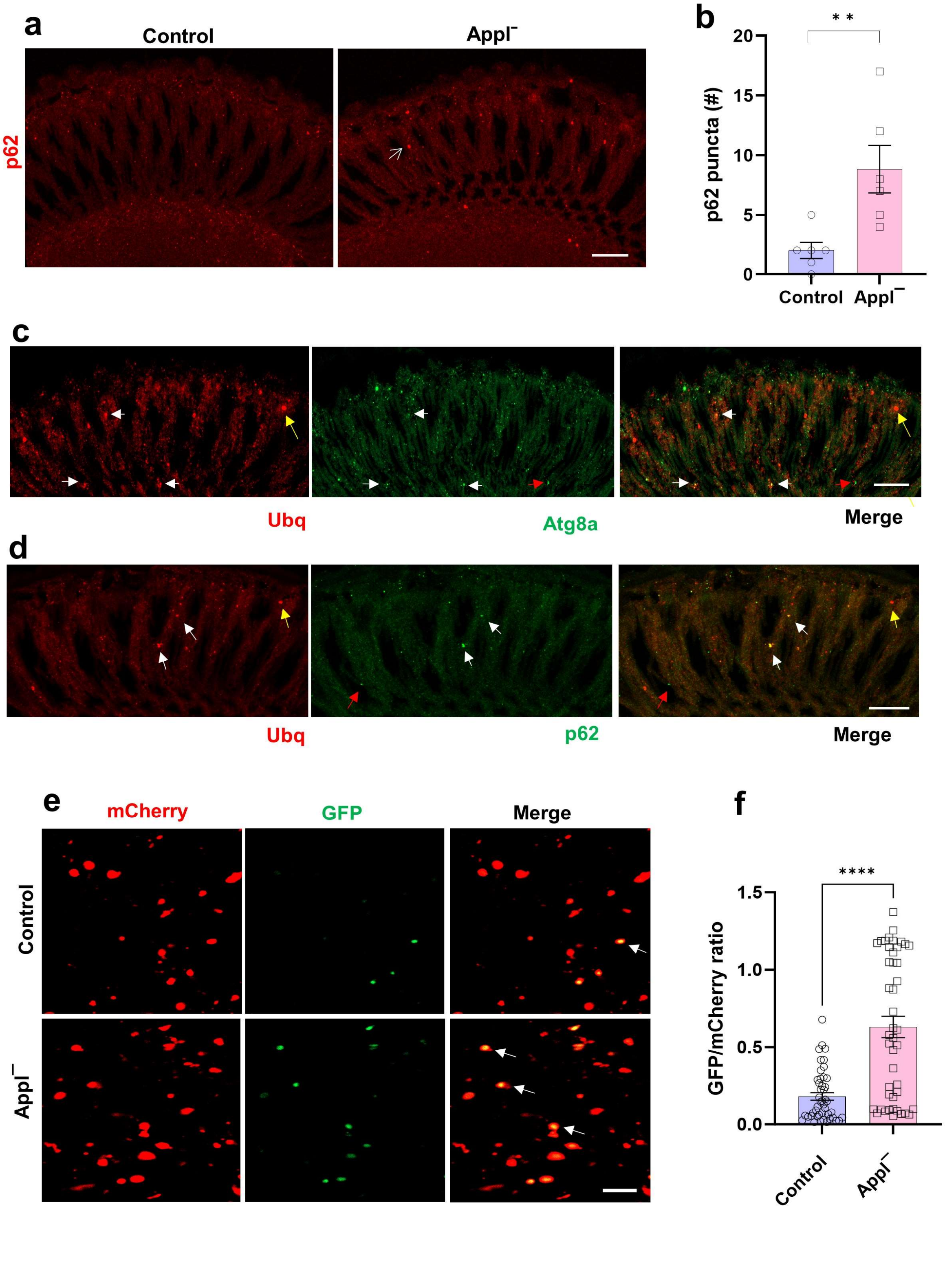

Supplementary Figure 4: Autophagy markers in flies with Appl loss. a Representative images illustrate increase in p62-positive aggregates (arrow) in the retina of *Appl*$^{-}$ mutant compared to control. **b** Quantification demonstrates significant increase of retinal p62-immunoreactive aggregates in retinas of *Appl*$^{-}$ mutants compared to controls. n = 6 per genotype. p = 0.008. **c,d** Ubiquitin-positive aggregates (red) occasionally colocalize with the Atg8a and p62 (green) in *Appl*$^{-}$ flies. White arrows indicate ubiquitin-positive aggregates colocalized with Atg8a **(c)** or p62 **(d).** Yellow arrows indicate ubiquitin-positive aggregates negative for Atg8a **(c)** or p62 **(d).** Red arrows indicate puncta positive for Atg8a **(c)** or p62 **(d)** and negative for ubiquitin. **e** Representative images of brains from control and *Appl*$^{-}$ flies expressing the tandem reporter GFP-mCherry-Atg8a. Arrows indicate GFP and mCherry double-positive puncta. **f** Quantification shows an increase in the ratio of GFP to mCherry fluorescence in the brains of *Appl*$^{-}$ flies, indicating impaired autophagic flux. n = 45 puncta from 3 animals per genotype. p = 1.086E-07. Control, *nSyb-GAL4/+* **(a,b)** and *UAS-GFP-mCherry-Atg8a/+; nSyb-GAL4/+* **(e,f).** **p<0.01, **** p < 0.0001, two-tailed Student's t-test. Data are represented as mean ± SEM. Scale bars, 20 μm **(a,c,d),** and 5 μm **(e).** Flies are 10 days old. Source data are provided as a Source Data file.

Supplementary Figure 5

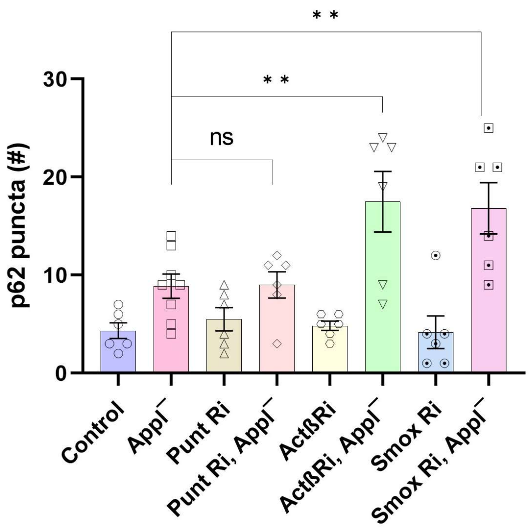

Supplementary Figure 5: Reduction in TGFß components increases p62-positive puncta in *Appl*¯ flies. Quantification of retinal p62-immunoreactive aggregates in flies with neuronal transgenic RNAi knockdown of TGFß pathway genes. Control is *nSyb-GAL4/*+. **p value 0.002, one-way ANOVA with Student-Newman-Keuls posthoc test. Data are represented as mean ± SEM. n = 6 per genotype. Flies are 10 days old. Source data are provided as a Source Data file.

Supplementary Figure 6

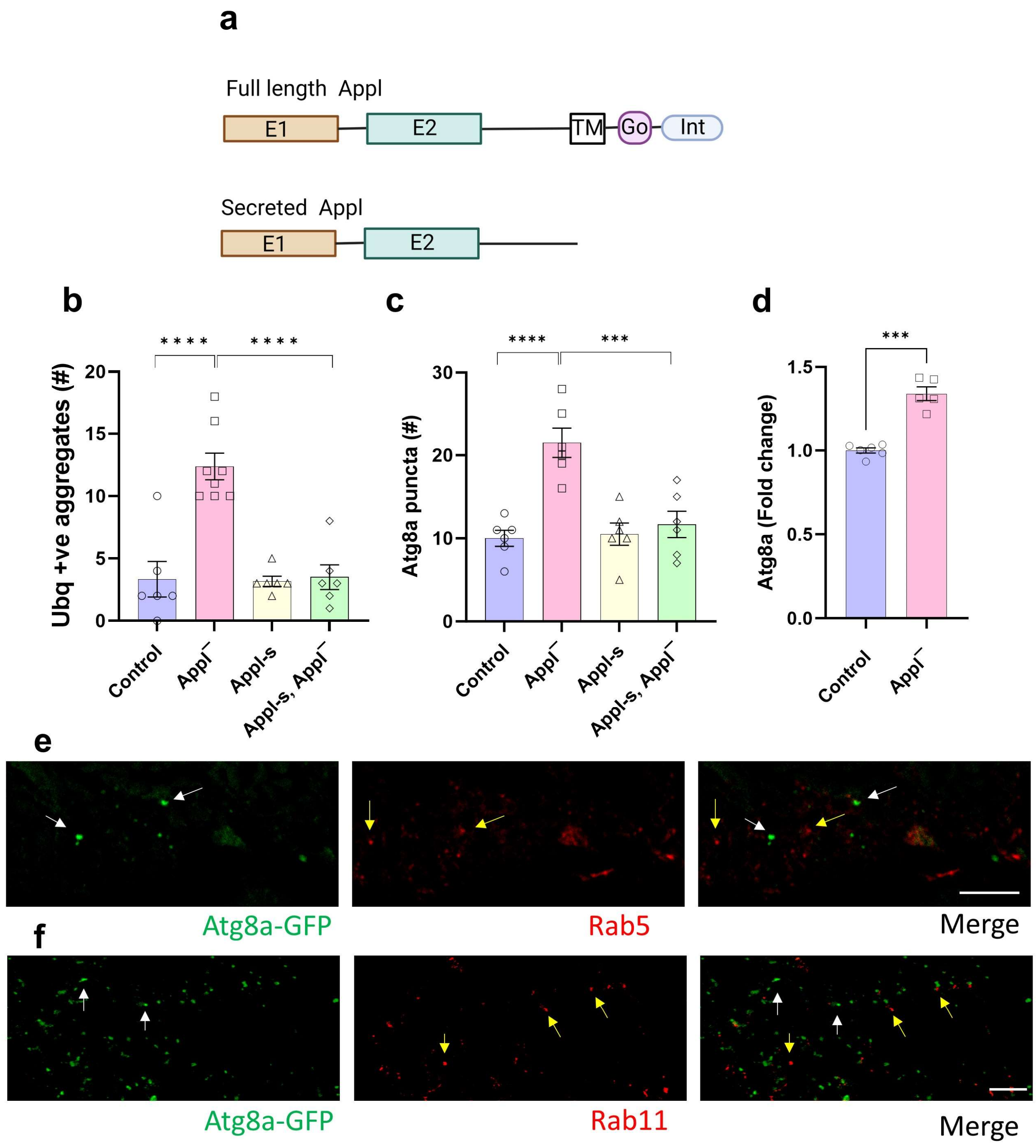

Supplementary Figure 6: Secreted Appl rescues proteostasis defects caused by the loss of Appl. a Schematic of full length Appl and secreted form expressed in (**b,c**). **b,c** Quantitative analyses show rescue of the increased numbers of ubiquitin-positive aggregates (**b**) or Atg8a-immunoreactive puncta (**c**) present in the retinas of *Appl* mutant flies by neuronal expression of

secreted Appl (Appl-s). In **b,** Control, n=6; *Appl⁻*, n=8; Appl-s, n = 6; Appl-s, *Appl⁻,* n=6. *In **c,** n=6 per genotype.* In **b**, p-values for Control vs *Appl⁻* and *Appl⁻* vs Appl-s, *Appl⁻* are < 0.0001. In **c**, p-values for Control vs *Appl⁻* and *Appl⁻* vs Appl-s, *Appl⁻* are < 0.0001 and 0.0002, respectively. **d** Mass spectrometric analysis using isobaric tandem mass tags (TMT) shows an increase in the protein level of the autophagy protein, Atg8a, in *Appl⁻* fly heads. Control, n = 6. *Appl⁻*, n = 5. p value = 0.0005. **e,f** Atg8a-GFP (white arrows, green) does not colocalize with the early endosomal marker, Rab5 (yellow arrows, red) or the recycling endosomal marker, Rab11 (yellow arrows, red). Control is *nSyb-GAL4/+* (**b,c**) and w^{1118} (**d**). Asterisks indicate *** p < 0.001, **** p < 0.0001, one-way ANOVA with Student-Newman-Keuls posthoc test (**b,c**) or, two-tailed Student's t-test (**d**). Data are represented as mean ± SEM. Scale bars, 5 µm. Flies are 10 days old. Source data are provided as a Source Data file.

Supplementary Figure 7

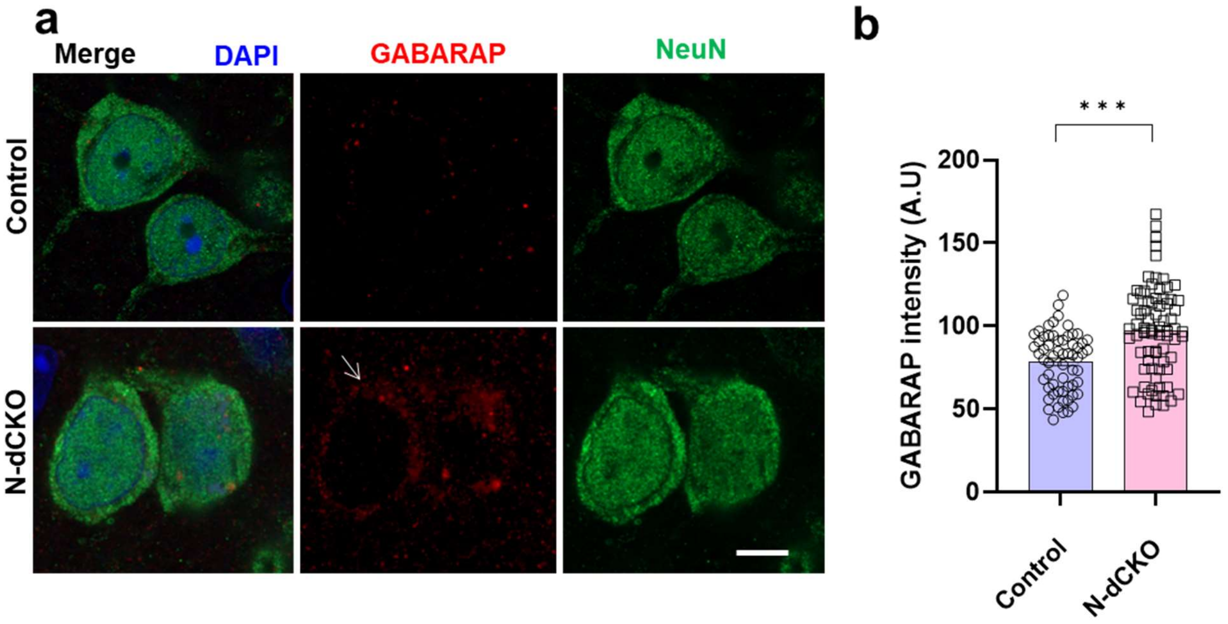

Supplementary Figure 7: Increased GABARAP in mice with loss of APP and APLP2 in neurons. a Representative immunofluorescence images show increased GABARAP (arrow, red) immunostaining in N-dCKO neurons (NeuN, green), additionally stained for DAPI (blue), compared to controls. **b** Quantitative analysis shows significantly increased GABARAP levels in N-dCKO neurons compared to controls. p value = 4.77E-06. Control is *APLP2*$^{-/-}$. ***p<0.0001, two-tailed Student's t-test. Data are represented as mean ± SEM. Control, n = 60 cells; N-dCKO, n = 75 cells. n = 5 animals per genotype. Scale bar is 5 µm. Mice are 18 months old. Source data are provided as a Source Data file.

Supplementary Figure 8

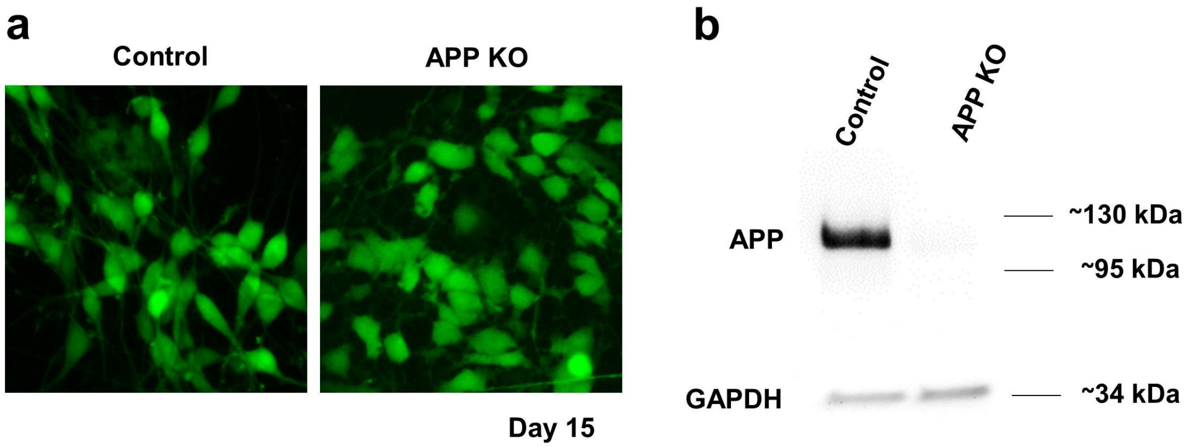

Supplementary Figure 8: Human iPSC-derived APP knockout neurons. a GFP is induced together with NGN2 and GFP fluorescence allows visualization of cellular morphology at day 15 following neuronal induction in iPSC-derived control and APP knockout neurons. **b** Western blot shows absence of detectable APP in knockout neurons. Knockout of APP is confirmed using three repeats. Source data are provided as a Source Data file.

Supplementary Figure 9

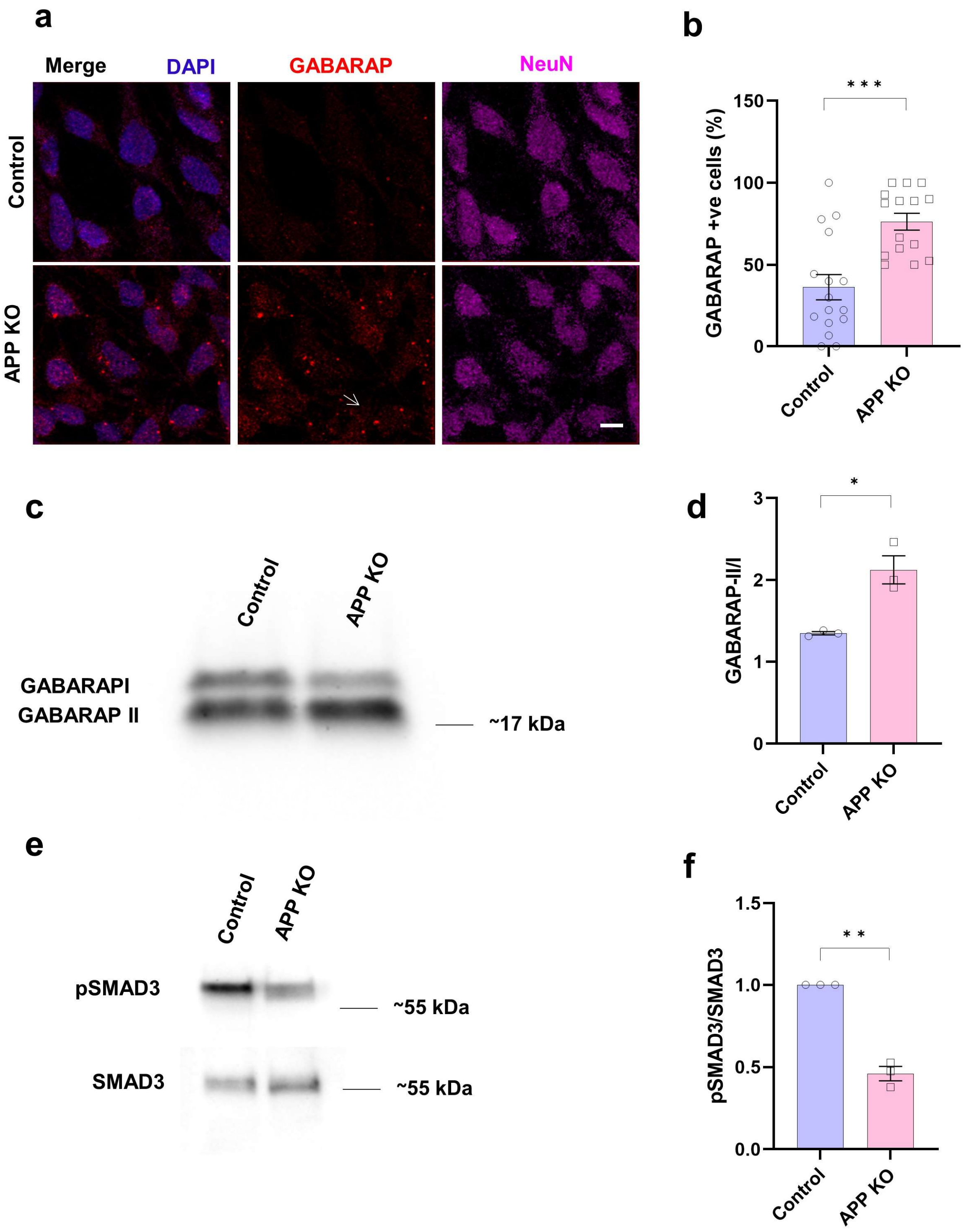

Supplementary Figure 9: Altered autophagy and TGFß pathway markers in human iPSC-derived APP knockout neurons. a Representative immunofluorescence images show increased GABARAP (arrow, red) immunostaining in APP knockout neurons (NeuN, magenta), additionally stained for DAPI (blue), compared to controls. **b** Quantitative analysis shows significantly increased GABARAP levels in APP knockout neurons compared to controls. Control, n = 16; APP KO, n = 15. p value = 0.0002. **c,d** Western blot (**c**) and quantification (**d**) show an increased in the GABARAP-II to I ratio in APP knockout neurons compared to controls. p value = 0.045. **e,f** Western blot (**e**) and quantification (**f**) show decreased phosphorylation of the TGFβ transcription SMAD3 in APP knockout neurons compared to controls. p value = 0.0065.*p<0.05, **p<0.01, ***p<0.001, two-tailed Student's t-test (**b**,**d**,**f**). Data are represented as mean ± SEM. Cells from 3 independent differentiations of APP knockout and isogenic control cells are tested. (**b**), n = 3 (**d,f**). Scale bar is 5 µm. Source data are provided as a Source Data file.

Supplementary Figure 10

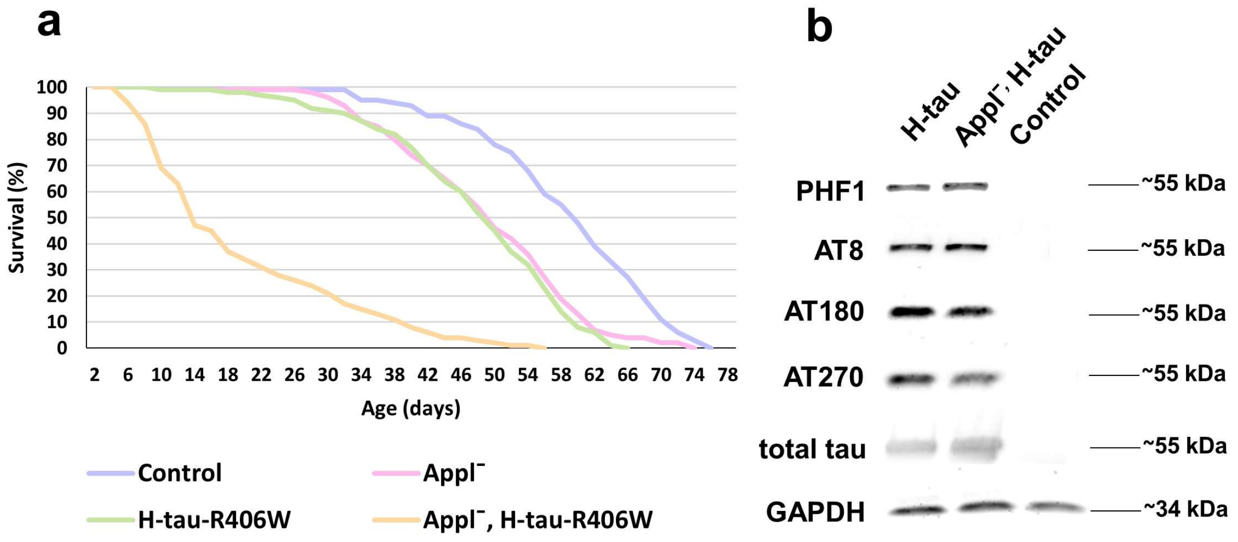

Supplementary Figure 10: Loss of Appl reduces longevity in *Drosophila* tauopathy model without clearly altering transgenic tau levels or phosphorylation. a Reduced lifespan in flies expressing R406W mutant human tau in an *Appl⁻* mutant background. FTDP-17 linked human mutant R406W tau was used in lifespan studies together with the *elav-GAL4* driver because lifespan truncation is robust in these animals.[15] n = at least 350 per genotype. Control is *elav-GAL4*/+. **b** Western blot shows no clear alteration in human wild type tau phosphorylation or total human wild type tau (phosphorylation-independent antibody) levels in *Appl⁻* flies expressing wild type human tau. Data from two independent blots are included. Control is *nSyb-GAL4*/+. Western blotting was performed on 1-day-old flies. Source data are provided as a Source Data file.
