# Supplementary material for: Integrative analysis reveals a conserved role for the amyloid precursor protein in proteostasis during aging
Source: Nat Commun. 2023 Nov 3;14:7034. doi: 10.1038/s41467-023-42822-1 (PMC10624868; doi:10.1038/s41467-023-42822-1)
Supplement: Supplementary file 4 — Reporting Summary [file 41467_2023_42822_MOESM4_ESM.pdf]

nature portfolio

Corresponding author(s): Mel B. Feany

Last updated by author(s): Oct 6, 2023

Reporting Summary

Nature Portfolio wishes to improve the reproducibility of the work that we publish. This form provides structure for consistency and transparency in reporting. For further information on Nature Portfolio policies, see our Editorial Policies and the Editorial Policy Checklist.

Statistics

For all statistical analyses, confirm that the following items are present in the figure legend, table legend, main text, or Methods section.

n/a	Confirmed	
☐	☒	The exact sample size (*n*) for each experimental group/condition, given as a discrete number and unit of measurement
☐	☒	A statement on whether measurements were taken from distinct samples or whether the same sample was measured repeatedly
☐	☒	The statistical test(s) used AND whether they are one- or two-sided<br>*Only common tests should be described solely by name; describe more complex techniques in the Methods section.*
☒	☐	A description of all covariates tested
☐	☒	A description of any assumptions or corrections, such as tests of normality and adjustment for multiple comparisons
☐	☒	A full description of the statistical parameters including central tendency (e.g. means) or other basic estimates (e.g. regression coefficient) AND variation (e.g. standard deviation) or associated estimates of uncertainty (e.g. confidence intervals)
☐	☒	For null hypothesis testing, the test statistic (e.g. F, t, r) with confidence intervals, effect sizes, degrees of freedom and P value noted<br>*Give P values as exact values whenever suitable.*
☒	☐	For Bayesian analysis, information on the choice of priors and Markov chain Monte Carlo settings
☒	☐	For hierarchical and complex designs, identification of the appropriate level for tests and full reporting of outcomes
☒	☐	Estimates of effect sizes (e.g. Cohen's *d*, Pearson's *r*), indicating how they were calculated

Our web collection on statistics for biologists contains articles on many of the points above.

Software and code

Policy information about availability of computer code

Data collection	Zeiss Zen
Data analysis	GraphPad Prism 9, FlyEnrichr, Geneontology.org, STRING, BioRender, ImageJ, DRSC scRNA-seq database, single cell data were visualized using uniform manifold approximation and projection (UMAP). Seurat, R package, was used for cell clustering

For manuscripts utilizing custom algorithms or software that are central to the research but not yet described in published literature, software must be made available to editors and reviewers. We strongly encourage code deposition in a community repository (e.g. GitHub). See the Nature Portfolio guidelines for submitting code & software for further information.

Data

Policy information about availability of data

All manuscripts must include a data availability statement. This statement should provide the following information, where applicable:

- Accession codes, unique identifiers, or web links for publicly available datasets
- A description of any restrictions on data availability
- For clinical datasets or third party data, please ensure that the statement adheres to our policy

The proteomics and ubiquitinomics data generated in this study have been deposited in the ProteomeXchange Consortium under accession code PXD041862 [https://www.proteomexchange.org/]. The single-cell RNA sequencing data generated in this study have been deposited in the Gene Expression Omnibus (GEO) repository under accession code GSE231518 [http://www.ncbi.nlm.nih.gov/geo/]. Single cell RNA

seq data, proteomics, and ubiquitinomics data are made publicly accessible. Publicly available DRSC scRNA-seq DataBase [https://www.flyrnai.org/tools/single_cell/web/] was used for annotation of the cell clusters. Source data are provided with this paper.

Research involving human participants, their data, or biological material

Policy information about studies with human participants or human data. See also policy information about sex, gender (identity/presentation), and sexual orientation and race, ethnicity and racism.

Reporting on sex and gender	Not Applicable
Reporting on race, ethnicity, or other socially relevant groupings	Not Applicable
Population characteristics	Not Applicable
Recruitment	Not Applicable
Ethics oversight	Not Applicable

Note that full information on the approval of the study protocol must also be provided in the manuscript.

Field-specific reporting

Please select the one below that is the best fit for your research. If you are not sure, read the appropriate sections before making your selection.

☒ Life sciences ☐ Behavioural & social sciences ☐ Ecological, evolutionary & environmental sciences

For a reference copy of the document with all sections, see nature.com/documents/nr-reporting-summary-flat.pdf

Life sciences study design

All studies must disclose on these points even when the disclosure is negative.

Sample size	Sample size was determined according to the standards in the field. No power analysis was performed. Number of animals used in this study are consistent with other publications in the field such as https://doi.org/10.1038/s41467-023-38421-9, https://doi.org/10.1038/ncomms3042, https://doi.org/10.1038/ncomms14161, https://doi.org/10.1523/JNEUROSCI.1429-16.2016.
Data exclusions	No data point was excluded
Replication	Data represents 3-6 biological replicates. All attempts at the replication were successful.
Randomization	For each replicate in an experimental group, fixed number of samples were randomly chosen. Samples were not alloted into experimental groups randomly. They were alloted based on their genotype.
Blinding	Investigators were not blinded as the biological groups were well defined and processed in parallel.

Reporting for specific materials, systems and methods

We require information from authors about some types of materials, experimental systems and methods used in many studies. Here, indicate whether each material, system or method listed is relevant to your study. If you are not sure if a list item applies to your research, read the appropriate section before selecting a response.

Materials & experimental systems

n/a	Involved in the study	
☐	☒	Antibodies
☐	☒	Eukaryotic cell lines
☒	☐	Palaeontology and archaeology
☐	☒	Animals and other organisms
☒	☐	Clinical data
☒	☐	Dual use research of concern
☒	☐	Plants

Methods

n/a	Involved in the study	
☒	☐	ChIP-seq
☒	☐	Flow cytometry
☒	☐	MRI-based neuroimaging

Antibodies

Antibodies used

ubiquitin (1:1000, P4G7, BioLegend; 1:200 Cell Signaling; 1:200, Z0458, Dako), GABARAP (1:1,000, endogenous Drosophila Atg8a, E1J4E, Cell Signaling; 1:200, Abcam), LC3B (1:200, Novus Biologicals), ref(2)P (1:600, Sarkar et al.,34), Rab5 (1:100, ab31261, Abcam), Rab11 (1:100, 610656, BD Biosciences), GFP (1:200, N86/8, NeuroMab), cleaved PARP (1:5000, E51, Abcam), elav (1:5, 9F8A9, Developmental Studies Hybridoma Bank), NeuN (1:400, EMD Millipore), MAP2 (1:100, EMD Millipore), pSMAD3 (1:200, Abcam). Alexa Fluor 488, 555, 647 (1:200, Invitrogen), Ubq (1:5000, P4D1, Cell Signaling; 1:5000, P4G7-HRP, BioLegend), Actin (1:10,000, Developmental Studies Hybridoma Bank), pSMAD3 (1:5000, ab52903, Abcam), SMAD3 (1:5000, ab40854, Abcam), pSMAD1/5/9 (1:2000, Cell Signaling), GABARAP (1:2000, Abcam), APP (1:1000, Sigma). PHF1 (1:50,000, gift from Peter Davies), AT8 (1:10,000, Thermo), AT180 (1:50,000, Thermo), AT270 (1:10,000, Thermo), total tau (1:75,000, A0024, Dako), GAPDH (1:20,000, Invitrogen).

Validation

We used antibodies under the conditions recommended by the manufactures. Statement of validation for the antibodies are given in the manufacturers website or in the previous publications mentioned here.

pSMAD3, ab52903: https://www.abcam.com/products/primary-antibodies/smad3-phospho-s423--s425-antibody-ep823y-ab52903.html.
SMAD3, ab40854: https://www.abcam.com/products/primary-antibodies/smad3-antibody-ep568y-ab40854.html.
pSMAD1/5/9: https://www.cellsignal.com/products/primary-antibodies/phospho-smad1-ser463-465-smad5-ser463-465-smad9-ser465-467-d5b10-rabbit-mab/13820.
APP, a8717: https://www.sigmaaldrich.com/US/en/product/sigma/a8717.
Tau AT180: https://www.thermofisher.com/antibody/product/Phospho-Tau-Thr231-Antibody-clone-AT180-Monoclonal/MN1040.
Tau AT270: https://www.thermofisher.com/antibody/product/Phospho-Tau-Thr181-Antibody-clone-AT270-Monoclonal/MN1050.
Ubiquitin, P4G7: https://www.biolegend.com/en-us/search-results/purified-anti-ubiquitin-antibody-13719?GroupID=ImportedGROUP1.
Ubiquitin, P4D1: https://www.cellsignal.com/products/primary-antibodies/ubiquitin-p4d1-mouse-mab/3936.
LC3, E1J4E: https://www.cellsignal.com/products/primary-antibodies/gabarap-e1j4e-rabbit-mab/13733.
GABARAP, ab109364: https://www.abcam.com/products/primary-antibodies/gabarapgabarapllgabarapl2-antibody-epr4805-ab109364.html.
LC3B, NB100-2220: https://www.novusbio.com/products/lc3b-antibody_nb100-2220.
Elav, 9F8A9: https://dshb.biology.uiowa.edu/Elav-9F8A9.
NeuN, MAB377: https://www.emdmillipore.com/US/en/product/Anti-NeuN-Antibody-clone-A60,MM_NF-MAB377.
MAP2, AB5622: https://www.emdmillipore.com/US/en/product/Anti-Microtubule-Associated-Protein-2-MAP2-Antibody,MM_NF-AB5622.
Tau, AT8: https://www.thermofisher.com/antibody/product/Phospho-Tau-Ser202-Thr205-Antibody-clone-AT8-Monoclonal/MN1020.
GFP, N86-8: https://dshb.biology.uiowa.edu/N86-8.
Actin, JLA20: https://dshb.biology.uiowa.edu/JLA20.
PHF1: doi.org/10.1038/78078.
Ref(2)P (Reference 34).
Rab5, ab31261: https://www.abcam.com/products/primary-antibodies/rab5-antibody-drosophila-early-endosome-marker-ab31261.html.
PARP, ab32064: https://www.abcam.com/products/primary-antibodies/cleaved-parp1-antibody-e51-ab32064.html (https://doi.org/10.1016/j.neuron.2017.11.036).
Ubiquitin, z0458: https://www.citeab.com/antibodies/3382935-z0458-ubiquitin (doi: 10.1073/pnas.1419083111).
Rab11, 610656: https://www.bdbiosciences.com/en-us/products/reagents/microscopy-imaging-reagents/immunohistochemistry-reagents/purified-mouse-anti-rab11.610656 (doi: 10.1371/journal.pgen.1008626)

Eukaryotic cell lines

Policy information about cell lines and Sex and Gender in Research

Cell line source(s)

NGN2 inducible iPSCs derived from female were obtained from Brigham and Women's Hospital iPSC NeuroHub

Authentication

APP knockout was tested using western blot

Mycoplasma contamination

Cell lines were not tested for mycoplasma contamination.

Commonly misidentified lines
(See ICLAC register)

Commonly misidentified cell lines were not used in this study

Animals and other research organisms

Policy information about studies involving animals; ARRIVE guidelines recommended for reporting animal research, and Sex and Gender in Research

Laboratory animals

Following Drosophila fly lines were used: nSyb-GAL4, elav-GAL4, UAS-punt-RNAi (TRiP.GL00069, TRiP.HMS01944), UAS-Actbeta-RNAi (TRiP.JF03276, GD3157), UAS-Smox-RNAi (TRiP.JF02320), UAS-Atg8a-GFP, Appld, UAS-APP.695.Exel, UAS-Appl.s. UAS-CD8-PARPVenus, UAS-GFP-mCherry-Atg8a. Age of the flies used are given in the figure legends. Ten days old flies were used for the omics analysis and immunostaining experiments. Twenty days old flies were used for climbing assay and neurodegeneration studies.

	Neuronal-APP conditional knockout mice were generated by crossing floxed APP mice with transgenic mice expressing Cre-recombinase under the neuronal nestin promoter. These neuronal conditional knockout mice were bred with APLP2 null mice to generate neuronal double conditional knockout (N-dCKO) mice. Backcrossings were performed with C57BL/6J strain for six generations. Above mentioned procedures were performed by the Zheng lab as described in doi: 10.1073/pnas.1012568107. Mice housing and feeding were not performed. Brains from 18-month-old mice were provided by the Zheng lab.
Wild animals	The study did not involve wild animals
Reporting on sex	Findings of this study do not apply to one specific gender. Therefore, gender related information was not collected for mice studies. Equal number of male and female flies were used for whole proteomics, ubiquitinomics, and single-cell RNA sequencing. As Appl is present in the X chromosome, male flies were used for genetic studies.
Field-collected samples	Study did not involves samples collected from the field
Ethics oversight	This study complies with all relevant ethical regulations.

Note that full information on the approval of the study protocol must also be provided in the manuscript.

Plants

Seed stocks	Not Applicable
Novel plant genotypes	Not Applicable
Authentication	Not Applicable
